# Heterogeneity in prevalence of subclinical *Plasmodium falciparum* and *Plasmodium vivax* infections but no parasite genomic clustering in the Chittagong Hill Tracts, Bangladesh

**DOI:** 10.1186/s12936-022-04236-0

**Published:** 2022-07-14

**Authors:** Tiffany Huwe, Mohammad Golam Kibria, Fatema Tuj Johora, Ching Swe Phru, Nusrat Jahan, Mohammad Sharif Hossain, Wasif Ali Khan, Ric N. Price, Benedikt Ley, Mohammad Shafiul Alam, Cristian Koepfli

**Affiliations:** 1 Department of Biological Sciences and Eck Institute for Global Health, University of Notre Dame, Notre Dame, USA; 2 Infectious Diseases Division, International Centre for Diarrhoeal Disease Research Bangladesh (Icddr, B), Dhaka, Bangladesh; 3 Georgia State University, Atlanta, GA USA; 4 Global and Tropical Health Division, Menzies School of Health Research and Charles Darwin University, Darwin, Australia; 5 Centre for Tropical Medicine and Global Health, Nuffield Department of Clinical Medicine, University of Oxford, Oxford, UK; 6 Mahidol-Oxford Tropical Medicine Research Unit (MORU), Faculty of Tropical Medicine, Mahidol University, Bangkok, Thailand

**Keywords:** Malaria, *Plasmodium falciparum*, *Plasmodium vivax*, Bangladesh, Chittagong Hill Tracts, Pahari, Ethnic groups, Genotyping, Population structure, Clustering

## Abstract

**Background:**

Malaria remains endemic in Bangladesh, with the majority of cases occurring in forested, mountainous region in the Chittagong Hill Tracts (CHT). This area is home to Bengali and diverse groups of indigenous people (Pahari) residing largely in mono-ethnic villages.

**Methods:**

1002 individuals of the 9 most prominent Pahari and the Bengali population were randomly selected and screened by RDT and qPCR. Parasites were genotyped by *msp2* and deep sequencing of 5 amplicons (*ama1-D3, cpmp, cpp, csp*, and *msp7*) for *Plasmodium falciparum* (n = 20), and by microsatellite (MS) typing of ten loci and amplicon sequencing of *msp1* for *Plasmodium vivax* (n = 21). Population structure was analysed using STRUCTURE software. Identity-by-state (IBS) was calculated as a measure of parasite relatedness and used to generate relatedness networks.

**Results:**

The prevalence of *P. falciparum* and *P. vivax* infection was 0.7% by RDT (*P. falciparum* 6/1002; *P. vivax* 0/1002, mixed: 1/1002) and 4% by qPCR (*P. falciparum* 21/1002; *P. vivax* 16/1002, mixed: 5/1002). Infections were highly clustered, with 64% (27/42) of infections occurring in only two Pahari groups, the Khumi and Mro. Diversity was high; expected heterozygosity was 0.93 for *P. falciparum* and 0.81 for *P. vivax*. 85.7% (18/21) of *P. vivax* and 25% (5/20) of *P. falciparum* infections were polyclonal. No population structure was evident for either species, suggesting high transmission and gene flow among Pahari groups.

**Conclusions:**

High subclinical infection prevalence and genetic diversity mirror ongoing transmission. Control activities should be specifically directed to Pahari groups at greatest risk.

**Supplementary Information:**

The online version contains supplementary material available at 10.1186/s12936-022-04236-0.

## Background

The Bangladesh National Malaria Control Programme has made significant progress towards controlling malaria within the country, reducing the incidence of malaria and mortality by 40% between 2015 and 2020 [1]. However, malaria remains hypoendemic in several eastern districts with an estimated 17 million people at risk [1]. The epidemiology of malaria in Bangladesh is complex, with four parasite species present. Infections with *Plasmodium falciparum* account for 86% of all clinical cases, *Plasmodium vivax for* 12%, and *Plasmodium malariae* and *Plasmodium ovale* account for remaining cases [1]. The spatial distribution of clinical cases is highly heterogeneous, with the greatest incidence recorded in the mountainous, forested Chittagong Hill Tracts (CHT) encompassing Bandarban, Rangamati, and Khagrachhari districts in the southeastern part of the country neighbouring Myanmar and India. Despite this region contributing only 7% of the national population, approximately 90% of malaria cases in Bangladesh are reported from the CHT [2]. Distribution of malaria is heterogeneous within the CHT as well, with 85% of malaria cases attributable to 11% of the population at risk for malaria [2].

The CHTs are home to at least 12 diverse indigenous groups, collectively known as Pahari, as well as increasing numbers of non-indigenous Bengali. Specific risk factors for malaria include jhum cultivation (the practice of forest-based shifting cultivation) and residing close to dense forest and at higher elevations [3–5]. The risk of malaria and subclinical *P. falciparum* infection vary among Pahari groups, likely due to differences in lifestyle and geographic location [3, 5, 6].

To ensure ongoing progress towards malaria elimination, it is critical that malaria control efforts are targeted to those at the greatest risk of infection, including parasite reservoirs that sustain ongoing transmission [7, 8]. Subclinical infections represent a key challenge for malaria elimination, as they are not identified by passive detection strategies. For instance, in a mass survey of tribal communities in the Indian Balaghat district, slide positivity rates of 32.4% and 29.0% were reported for febrile and afebrile individuals, respectively [9]. Furthermore, in nearly all transmission settings, many subclinical infections are below the limit of detection of microscopy or rapid diagnostic test [10, 11]. A survey in the CHT conducted between 2009 and 2012 reported a 1% prevalence of subclinical *P. falciparum* infection by light microscopy, which persisted year-round [5]. The true infection rate was likely higher; more sensitive molecular methods, such as qPCR, are required to detect these infections. The prevalence of subclinical infections and associated risk factors have been well described for regions where the transmission intensity of *P. falciparum* and *P. vivax* is moderate or high; however, few studies have been conducted in low endemic, pre-elimination settings, and in regions where *P. malariae* and *P. ovale* are also present.

Parasite genotyping is being applied increasingly to provide a better understanding of residual transmission [12–14]. If infections among villages or at-risk groups are closely related and separated from other groups, most transmission is predicted to occur within these groups. In contrast, high parasite genetic diversity and absence of population structure indicate transmission among groups [13, 15–17].

To understand transmission patterns and differences in infection prevalence, residents of the CHT were enrolled into a cross sectional survey and blood samples were screened for any *Plasmodium* spp. infection using qPCR [18]. Genotyping by size-polymorphic markers and amplicon deep-sequencing was applied to assess relationships among infections within and between ethnic groups.

## Methods

### Ethics statement

The study obtained ethical approval from the Ethics Review Committee of the icddr,b, Bangladesh (PR-15021), the Human Research Ethics Committee of the Northern Territory Department of Health and Menzies School of Health Research, Australia (HREC 2015–2336), and the University of Notre Dame Institutional Review Board (18-09-4875). Written informed consent was collected from all participants or their legal guardians prior to enrollment and in addition, written assent was collected from all minors above the age of 11 years [19].

### Sample collection

**Fig. 1:**
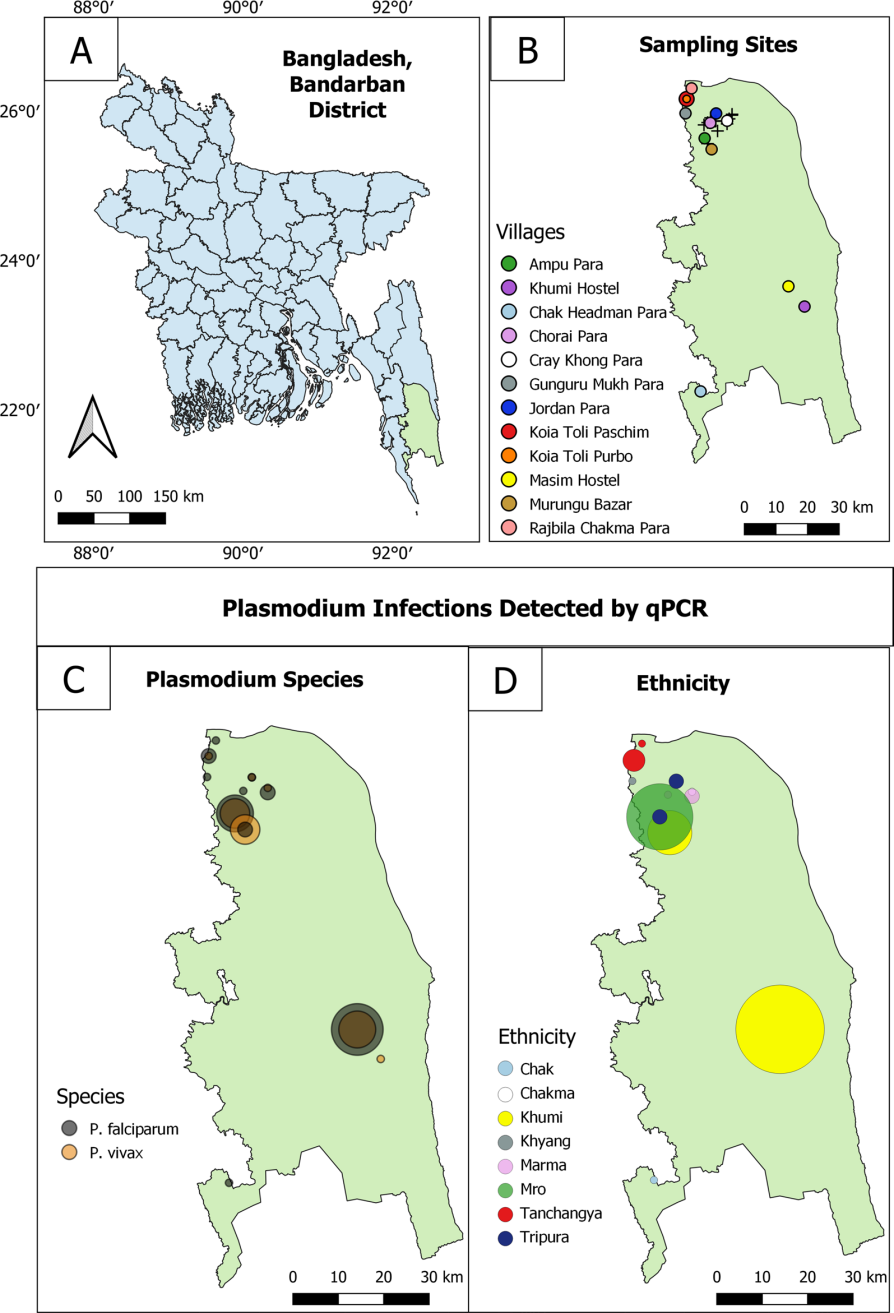
**A** Location of Bandarban District in Bangladesh. **B** Villages sampled for this study (24 of 28). Villages in which *Plasmodium* spp. samples were detected are labelled by colour. Villages in which no infections were detected are marked with a (+). Not pictured are Faruk Para, Hebron Para, Hati Bhanga Para, Paschim Antaha Para, and Purbo Antaha Para for which GPS location was not recorded. **C** Distribution of *Plasmodium* spp. infections detected by qPCR. *Plasmodium falciparum* and *P. vivax* were both found throughout the study area. **D** The majority of infections were clustered among the Khumi and Mro

As the aim of this study was to understand malaria epidemiology among different ethnic groups in the CHT, villages were purposively selected to represent different ethnicities [19]. Most villages in the CHT are mono-ethnic. Multiple villages were visited when necessary to sample roughly 100 individuals among the Bengali and each of the 9 targeted Pahari groups. The collection area spanned a range of approximately 150 km from the north to the south of the CHT (Fig. 1). Between August 2015 and January 2016 (overlapping with the June to October monsoon season), venous blood samples (5 mL) were collected in EDTA Vacutainers (BD, USA) from individuals aged more than five years old irrespective of the presence of symptoms of malaria. Blood for rapid diagnostic tests (RDTs) was collected by finger prick from the same individuals.

### Laboratory methods

DNA was extracted from 200 µL blood using the Qiagen DNeasy kit according to manufacturer’s instructions. 4 µL of DNA, corresponding to 4 µL of blood, were screened by qPCR for *P. falciparum* using the *varATS* assay [20], and for *P. vivax* using the *cox1* assay [21]. The *varATS* assay targets multicopy genes and amplifies approximately 20 copies per genome. *Cox1* is a mitochondrial gene and present in approximately 10 copies per genome. Samples were screened for *P. ovale* and *P. malariae* using single gene assays [22].

### Parasite genotyping

*Plasmodium falciparum*-positive samples were genotyped by size-polymorphic *msp2* and 5 highly diverse amplicons. *msp2* typing was done by nested PCR followed by capillary electrophoresis [23]. Amplicon deep sequencing was done for markers *apical membrane antigen 1* (*ama1-D3,* PF3D7_1133400), *circumsporozoite surface protein* (*csp*, PF3D7_0304600), *merozoite surface protein 7* (*msp7*, PF3D7_1335100), a conserved *Plasmodium* membrane protein (*cpmp*, PF3D7_0104100), and a conserved *Plasmodium* protein (*cpp*, PF3D7_1475800) [24].

*Plasmodium vivax*-positive samples were typed by 10 microsatellite markers (MS1, MS2, MS4, MS5, MS6, MS7, MS9, MS10, MS15, MS20) [25] and one amplicon (*Pvmsp1*). Peaks higher than 300 units of relative fluorescent intensity and above background noise were considered true clones. Peaks less than 1/3 the height of the highest peak were not recorded. Alleles were grouped using TANDEM software [26]. Amplicon deep sequencing was done for merozoite surface protein 1 (*Pvmsp1*, PVP01_0728900*)* as described [27] with the following modifications: the 5′ ends of the forward and reverse primers for the nested PCR included the overhang sequences 5′-GTGACCTATGAACTCAGGA-3′ and 5′-CTGAGACTTGCACATCGCAGC-3′, respectively.

PCR products were run on a 2% agarose gel, then 4uL of PCR product from each sample was combined to create pools of similar marker concentration as estimated from band intensities on the gel. These pools were purified using AMPure XP beads (Beckman Coulter) and DNA concentrations were measured using a Qubit fluorometer (Thermo Fisher Scientific). Pools were diluted to 0.1ug/uL and combined to form the sequencing library. The library was sequenced in paired-end mode in one run using the Illumina MiSeq reagent kit v3 600 cycle (2 × 300 bp) with 15% Enterobacteria phage *phiX* control v3 (Illumina).

*Plasmodium falciparum* positive samples were typed for *pfhrp2* deletion using a protocol based on droplet digital PCR (ddPCR) [28].

### Data analysis

Analyses were conducted using R software version 4.0.0 (Additional file 2) [29]. Haplotypes were determined using the bioinformatic pipeline from HaplotypR package version 0.3.3 [30]. Amplicon sequencing reads were demultiplexed by sample and marker. Overlapping paired end reads were merged via the vsearch package and clustered via the swarm package. Samples with ≤ 10 reads were excluded from further analysis. Single nucleotide polymorphisms were required to have a mismatch rate of at least 0.5 occurring in two or more samples for further consideration. Chimeric reads, singletons, and reads containing an insertion or deletion were excluded. Haplotype calling from the remaining reads required within-host haplotype frequency ≥ 1% and minimum coverage of 3 reads per haplotype and 25 reads per sample.

Statistical analyses were done in the R base package. Prevalence and parasitaemia were compared across age groups (5–10, 11–20, 21–30, 31–40, 41–50, 51–60, and > 60 years) by analysis of variance (ANOVA) and Tukey Post Hoc tests. Pearson’s Chi-squared tests were performed to test for differences in prevalence by sex, ethnicity, and village. Simple logistic regression was performed to separately compare participants’ village and ethnicity as predictors of infection. Multivariate regression was not performed due to high correlation between villages and ethnicities. Multiplicity of infection (MOI) for *P. falciparum* was defined as the maximum number of alleles detected for any one amplicon in a sample. MOI for *P. vivax* was defined as the number of alleles detected for the MS with the second most number of alleles to prevent overestimating MOI due to presence of PCR artifacts [31]. Expected heterozygosity (H_e_) and a modified identity-by-state (IBS) metric were calculated using R software version 3.6.3 (Company, Country) [32]. A modified IBS metric from Tessema, 2019 [32] was used to measure pairwise relatedness between infections based on all alleles detected, giving consideration to minor clones in polyclonal infections. For *P. falciparum*, IBS was calculated using data from the 5 amplicons. For *P. vivax,* IBS was calculated using data from the 10 MSs and the one amplicon. Structure plots were created with the rmaverick package in R, using no admixture and considering only the dominant clone in each sample. Relatedness networks were generated using R package igraph, with an IBS threshold of 0.5 for each link. This value is the theoretical relatedness of meiotic siblings; however, it is a less restrictive threshold than the more rigorous IBS ≥ 0.6 used for identification of highly related infections in some studies [33, 34]. Principal components analysis (PCA) was conducted using R package ade4 considering the dominant alleles at 5 amplicon markers for *P. falciparum* and at 1 amplicon and 10 MS markers for *P. vivax.*

## Results

### Prevalence of infection

In total, 1002 samples were collected from 28 villages, with 9–100 individuals sampled per village (Fig. 1, Additional file 1). 900 individuals were surveyed from 9 Pahari groups (99–101 individuals per group) as well as 100 individuals who identified as Bengali. Two individuals from a tenth Pahari group, the Lushai, were sampled in a majority Bawm village. Median age of participants was 31 years (range 5–80). Overall, 59.9% (600/1002) of individuals were female. Two individuals reported travel in the prior 4 weeks, one individual reported fever within the previous 48 h, and two individuals reported having had malaria in the last 90 days. These numbers were too low to assess them as risk factors for infection.

In total 42/1002 (4.2%) of individuals tested positive by qPCR for *P. falciparum* and/or *P. vivax* (21 *P. falciparum* mono-infection, 16 *P. vivax* mono-infection, 5 mixed) (Table 1). No *P. ovale* or *P. malariae* infections were detected. Only 7/1002 (0.70%) individuals tested positive by RDT or microscopy, one of which was not confirmed by qPCR. RDT and microscopy detected 6/26 *P. falciparum* infections and 1/5 mixed infections (Table 1). All individuals who tested positive by RDT or microscopy were aged 7 to 20 years.

**Table 1:** *Plasmodium* spp. infections detected by each diagnostic method

Diagnostic	*P. falciparum*	*P. vivax*	Mixed	Total
qPCR	21	16	5	42
RDT	6	0	1	7
Microscopy	3	0	1	4

Many more infections were detected by qPCR than by RDT or microscopy

One *P. falciparum* infection detected by RDT was not confirmed by qPCR

Prevalence by qPCR was highest in individuals aged between 11 and 20 years (7.69%, 16/208), and lowest in individuals older than 60 years (1.25%, 1/80). The only significant difference was between the 11–20 years and 21–30 years (1.62%, 3/185) age groups (ANOVA: *p* = 0.045; Tukey Post Hoc: *p* = 0.043) (Table 2). The prevalence in men was 5.97%, (24/402) compared to 3.00% (18/600) in females (*p* = 0.021). Parasitaemia did not differ among age groups for either *P. falciparum* (*p* = 0.545) or *P. vivax* (*p* = 0.749).

**Table 2:** Age distribution of participants with *Plasmodium* spp. Infections

Age (yrs)	n	Infections	Prevalence (%)
5–10	98	5	5.10
11–20	208	16	7.69
21–30	185	3	1.62
31–40	207	8	3.86
41–50	143	4	2.80
51–60	81	5	6.17
> 60	80	1	1.25
Total	1002	42	

The highest prevalence was in the 11–20 age group (7.69%) and the lowest prevalence was in the > 60 age group (1.25%)

There were significant differences in the prevalence of parasitaemia between ethnic groups (*P* < 0.0001), with 0% (0/100) of Bengali participants infected and 64% (27/42) infections concentrated among two of the ten Pahari groups (Table 3). In total, 18% (18/100) of Khumi and 9% (9/100) of Mro participants were infected. Among the other 8 ethnic groups, less than 4 individuals had parasitaemia detected by qPCR in each group. The differences in ethnicity were not reflected in RDT results. Among 10 Pahari groups, one or two positive RDTs were recorded in five groups.

**Table 3:** Distribution of *Plasmodium* spp. infections among ethnicities as detected by qPCR and RDT

	qPCR				RDT
Ethnicity	*P. vivax*	*P. falciparum*	Mixed	Positive (%)	Positive (%)
Khumi	9	7	2	18	1
Mro	4	4	1	9	1
Tripura	1	2	1	4	0
Tanchangya	0	3	1	4	2
Marma	0	3	0	3	2
Chak	1	1	0	2	0
Chakma	1	0	0	1	0
Khyang	0	1	0	1	1
Bawm	0	0	0	0	0
Lushai	0	0	0	0	0
Bengali	0	0	0	0	0
Total	16	21	5	4.2	0.70

A much higher infection prevalence was detected by qPCR than by RDT

*Plasmodium vivax* and *P. falciparum* were detected in similar numbers (*Pv*: n = 16, *Pf*: n = 21, Mixed: n = 5)

Infections were highly clustered, with 64% occurring in the Khumi and Mro groups (27/42)

No infections were detected among Bengalis (0/100)

There were significant differences in the prevalence of parasitaemia between villages (*p* < 0.0001), with no infections detected in 50% (14/28) of villages. All Mro were sampled in one village (Ampu Para), where prevalence was 9% (9/100). Khumi were sampled in four villages, and prevalence was high in two of them: 12% (6/51) in Murungu Bazar, and 36% (12/33) Masim Hostel (Fig. 1).

Simple logistic regression identified two ethnicities as significant predictors of infection, Khumi (*p* < 0.0001) and Mro (*P* = 0.001), while village was not a significant predictor (*p* = 0.006).

### Parasite genotyping

All 21 individuals with *P. vivax* parasitaemia were successfully genotyped. Among 12 samples successfully typed by amplicon deep sequencing, the median coverage for amplicon marker *msp1* was 11,342 reads. Genetic diversity was high, with 5–11 alleles per MS marker (mean H_e_ = 0.81) and 7 alleles for *msp1*. MOI ranged from 1 to 3 (median = 2.0) and 85% (18/21) were polyclonal.

**Fig. 2:**
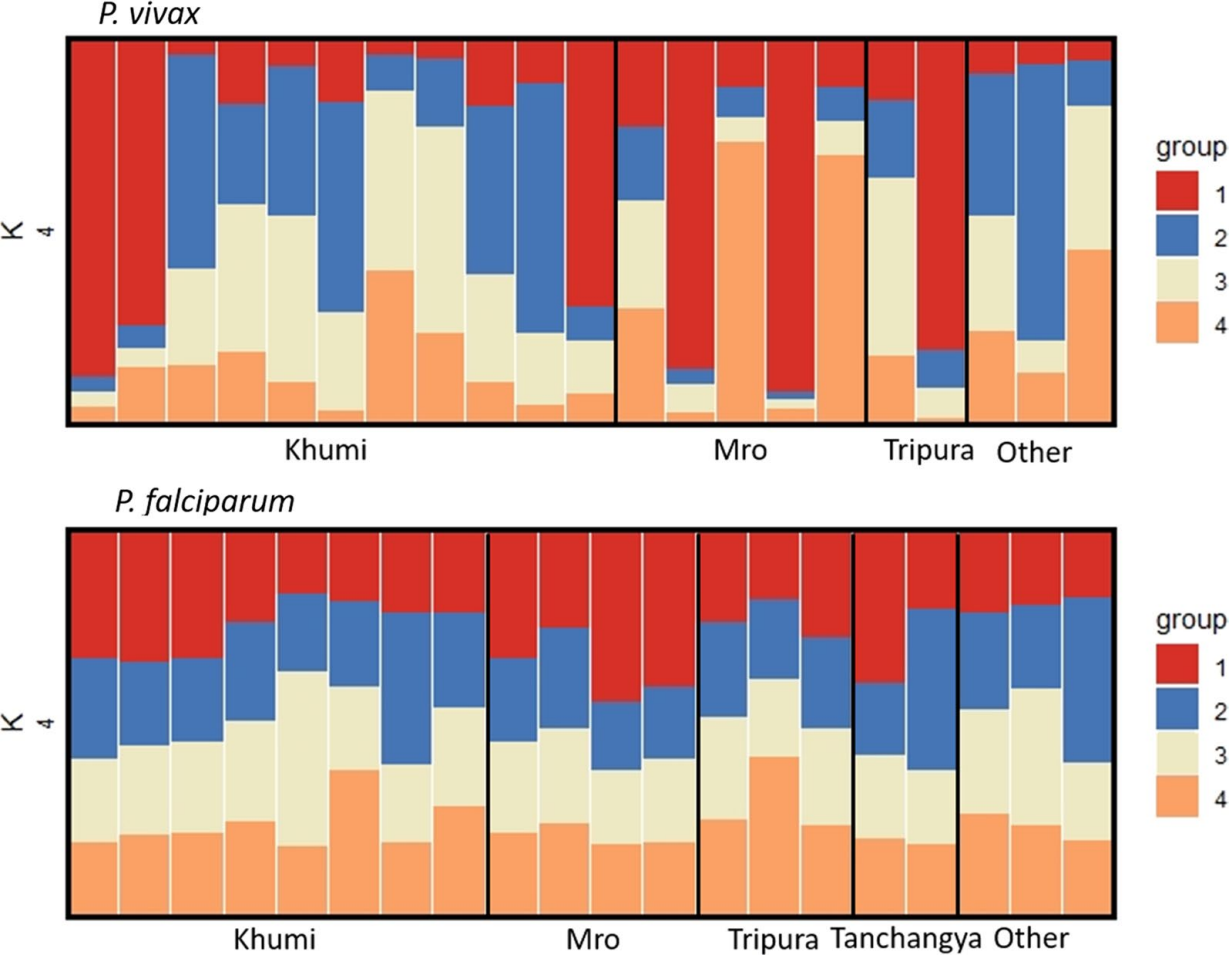
Structure plots showed no structure among *P. falciparum* or *P. vivax* populations. Plots were generated using k = 2–5, and all plots looked very similar

STRUCTURE plots were generated to infer population structure based on alleles of the major clones in each sample. Plots were generated using k = 2–5 as the majority of infection samples came from 2 Pahari groups, with less representation from a few other groups. There was no obvious population structure for *P. vivax* (Fig. 2).

The mean *P. vivax* IBS within ethnicities was 0.14 for the Khumi (n = 11), 0.31 for the Mro (n = 5), and 0.29 for Tripura (n = 2) (Table 4). No two samples had all 11 alleles in common. Relatedness networks were generated using a minimum threshold of IBS ≥ 0.5 for each link to generate the theoretical relatedness of meiotic siblings. Only one pair and one triplet of infections showed IBS > 0.5. These small networks included four of the five samples collected from Mro, and one Tripura sample. The Tripura sample was taken from the Hati Bhanga village adjacent to the Mro Ampu village from which all five Mro samples were collected.

**Table 4:** Identity-by-state among *Plasmodium* spp. samples

Ethnicity	n	Mean IBS
*P. vivax*		
Khumi	11	0.14
Mro	5	0.31
Tripura	2	0.29
All *P. vivax*	21	0.15
*P. falciparum*		
Khumi	8	0.12
Mro	4	0.50
Tripura	3	0.00
Tanchangya	2	0.05
All *P. falciparum*	20	0.11

Parasite relatedness within Pahari groups as measured by IBS. For both *P. vivax* and *P. falciparum*, mean IBS was greatest among parasites detected among the Mro

By PCA, *P. vivax* showed some separation of samples by ethnicity with the Khumi and Mro forming loose clusters, although the Khumi samples appeared evenly throughout most of the plot.

In total 76.9% (20/26) of individuals with *P. falciparum* parasitaemia were successfully genotyped by *msp2* size-polymorphic marker and 5 amplicons. The median coverage for amplicon markers was 14,729 reads (range 364–68,910, 2.5 and 97.5 percentiles) per amplicon marker and sample. Genetic diversity was high with 14 *msp2* alleles (3 FC27 and 11 3D7) and 8–15 alleles per amplicon marker (mean H_e_ = 0.93 across all amplicon markers)*.* The MOI ranged from 1 to 5 (median = 1) and 20% (4/20) of infections were polyclonal. None of the isolates had a *pfhrp2* deletion.

As for *P. vivax*, STRUCTURE analysis revealed no population structure for *P. falciparum* (Fig. 2), and none of *P. falciparum* isolates (with haplotypes called at more than 1 marker) had identical haplotypes. The mean IBS within ethnicities was 0.12 for the Khumi (n = 8), 0.50 for Mro (n = 4), 0.0 for Tripura (n = 3), and 0.05 Tanchangya (n = 2) (Table 4). Only the *P. falciparum* from the Mro had a mean IBS of at least 0.5, indicative of close relatedness.

**Fig. 3:**
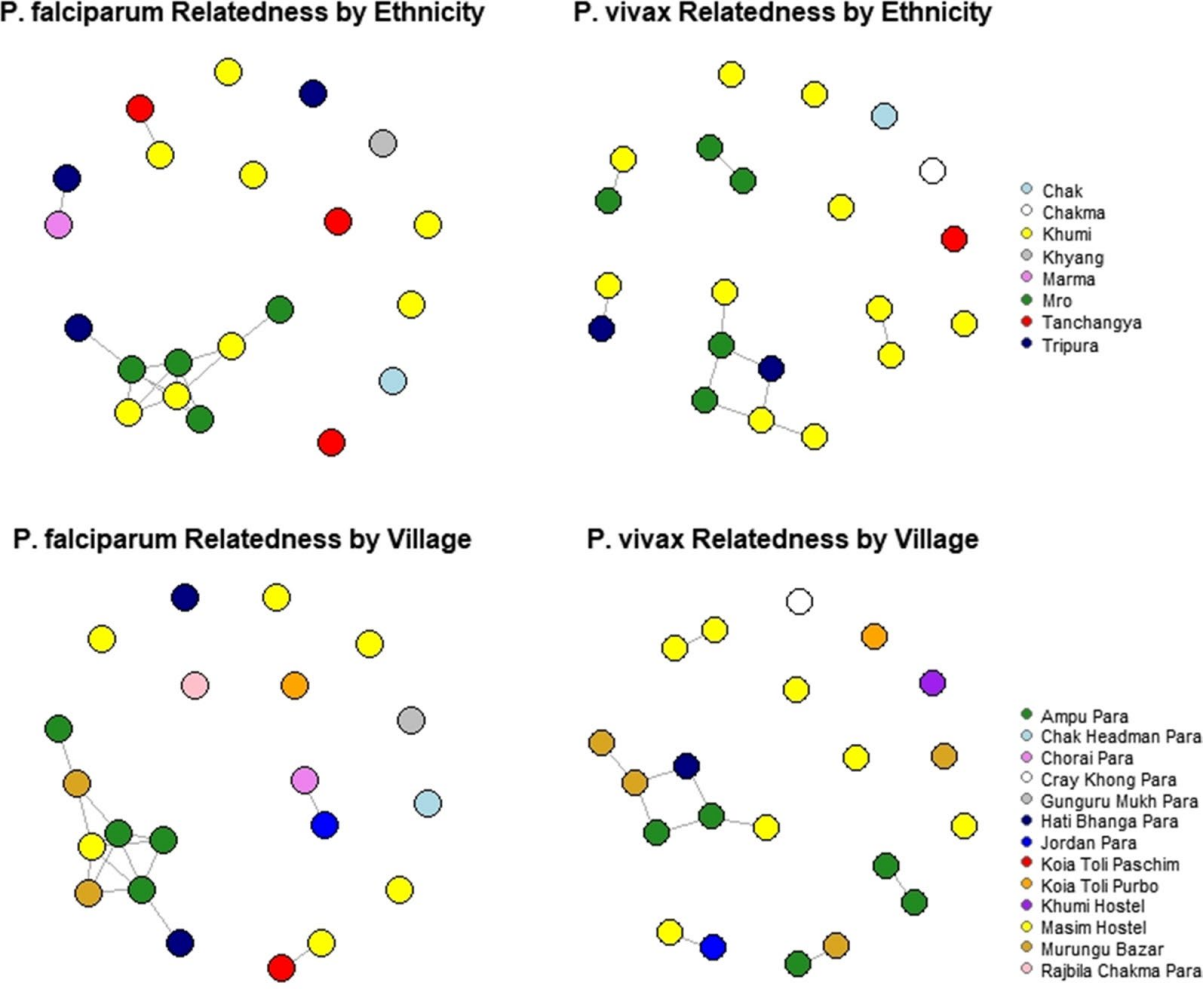
Relatedness networks based on pairwise IBS  ≥ 0.5. In the top row, samples are colored according to ethnicity; in the bottom row, samples are colored by village. Among *P. falciparum* there is a small network of related infections from the Khumi and Mro, plus one infection from the Tripura. Among *P. vivax* there are two smaller networks of related infections from the Mro plus one infection from the Tripura. There are very few linkages for either parasite species

*Plasmodium falciparum* infections showed a low level of clustering, with 40% (8/20) of isolates linked in a small network including samples from the Khumi (n = 3), Mro (n = 4), and Tripura (n = 1) (Fig. 3). Parasite isolates from Khumi individuals in this small network included 2/2 from the northern Murungu Bazar village and 1/6 from the southern village near Masim Hostel 75 km away. The two samples from Murungu Bazar were not linked to each other, but were connected in the network by the sample from Masim Hostel.

**Fig. 4:**
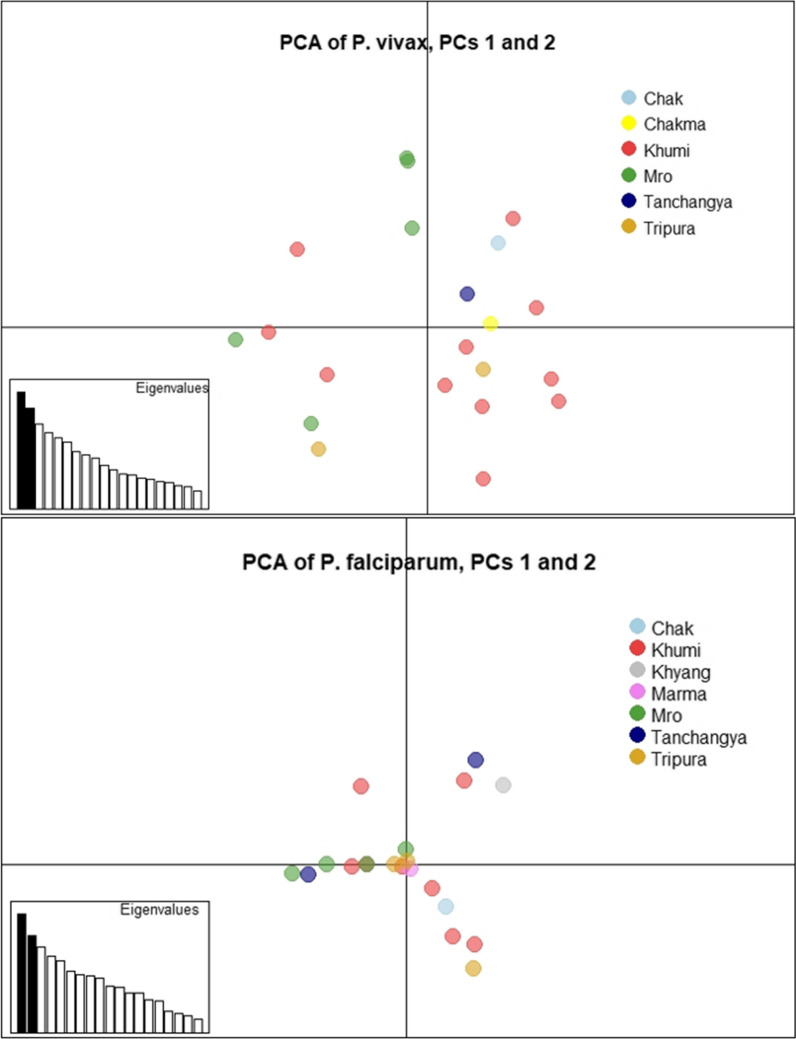
Principal components analysis based on principal components 1 and 2. *Plasmodium vivax* haplotypes showed some separation by ethnicity, with the Khumi and Mro forming loose clusters. *P. falciparum* samples showed very little separation, with samples appearing closely around the origin

By PCA, the *P. falciparum* haplotypes showed very little separation of samples, with samples appearing closely around the origin (Fig. 4). All 5 Mro samples clustered fairly close together, although they overlapped with samples from the Khumi, Tripura, and Tanchangya as well.

## Discussion

In the CHT in Bangladesh, infections were highly clustered among Pahari groups, with nearly two thirds of all infections occurring in just two out of ten Pahari groups surveyed, the Khumi (42%, 18/42) and Mro (21%, 9/42). The 1% overall prevalence measured by RDT in this study was the same as that measured by RDT and microscopy on samples collected from the CHT in 2009 to 2012 [5]. As observed in other low-transmission sites [35], the prevalence by qPCR was substantially higher than by RDT or microscopy.

There is significant spatial heterogeneity in the prevalence of infectious diseases, including that for malaria [36–38]. Where transmission intensity is low, control interventions are most  cost-effective if targeted towards those at greatest risk [53]; however, few studies have investigated the contribution of ethnic diversity to spatial heterogeneity [39, 40]. These findings suggest that clustering of infections is driven by ethnicity but not by village. *Plasmodium* spp. infections were far more prevalent among the Mro in Ampu Para and the Khumi in Murungu Bazar than in any of the nearby villages in northern Bandarban. Additionally, prevalence was elevated in a second Khumi village Masim Hostel, despite its location roughly 50 km to the south. In the dense forests of Indian subdistrict or Keshkal, a cross-sectional survey of three closely situated tribal groups demonstrated similar small-scale heterogeneity, with a significantly higher incidence of malaria in one group compared to the other two groups [41].

Despite the clustering of infections within ethnic groups, there was high genetic diversity and limited population structure for both *Plasmodium* species. Locally increased prevalence of infection in low transmission settings can arise from localized, small outbreaks, in which case, expansion of clonal or nearly related lineages would be expected [42–44]; however, this was not the case in this study in the CHT. The high diversity of parasite populations within villages and absence of population structure reflects ongoing transmission at a sufficiently high level to maintain genetic diversity, and that increased prevalence among certain ethnicities is not the result of within-village transmission of possibly imported cases. A similar pattern of high diversity and lack of population structure despite low transmission has also been observed in other sites, such as in Senegal, South Africa, and Eswanti [34, 45, 46], and suggests that local transmission is likely to be higher than previously predicted [46].

The causes for the pronounced differences in paras ite prevalence yet absence of any indication of small-scale transmission are unknown. Differences in behaviour, housing structure, and treatment-seeking are likely to play a role. A survey of health-seeking behaviour among five ethnic groups in the CHT in 2001 reported that the Mro had the lowest incidence of malaria (based on self-reported symptoms) and were the least likely to seek external healthcare when ill; this could reflect the greater distance to health facilities for the Mro [6]. Although this previous study did not survey any individuals from the Khumi group, both the Khumi and Mro may miss medical interventions and malaria control efforts due to their particular geographic isolation compared to other Pahari groups [6, 47]. Anecdotally, some Khumi believe that they have been excluded from government and NGO programmes aimed at Pahari due to lack of education or ability to speak Bangla [48]. National programmes are typically delivered in Bangla and sometimes with major Pahari languages e.g. Chakma and Marma [48]. An inability to communicate in these languages could present a major obstacle for seeking treatment and participating in vector control programmes. Future studies should consider social factors including participants’ bed net usage, distance to a health facility, and ability to communicate in Bangla. Alternatively, parasite heterogeneity may be attributable to human genetic factors resulting in a decreased risk of infection [49]. Lastly, stochastic effects owing to the inherent heterogeneity of infectious diseases should not be ruled out. A survey across multiple nearby villages in Papua New Guinea found prevalence of infection by PCR to range from 0 to 22% for *P. falciparum* and from 11 to 34% for *P. vivax* [50]. These villages were inhabited by the same ethnicities and similar in terms of housing and climate factors.

Although this study did not find any *pfhrp2* deletions in *P. falciparum* samples from the CHT, a deletion has been detected in Sylet in northeastern Bangladesh near the Indian border [51]. Additionally, deletions of *pfhrp2* and *pfhrp3* genes have been detected in northern India [52]. Continued surveillance for such gene deletions is warranted in this area.

## Conclusions

This study detected a substantial burden of subclinical *Plasmodium* infections clustered among the Khumi and Mro Pahari groups in the CHT. Infections were highly diverse and showed no population structure, indicating sustained transmission of parasites rather than a single, clonal outbreak. To ensure sustained progress towards malaria elimination in Bangladesh, the potential reservoir for ongoing transmission should be identified and targeted for public health control interventions.

## Supplementary Information

**Additional file 1:** Ethnicity, sex, age, and infection data for each participant; Haplotype data for *P. vivax* and *P. falciparum*.

**Additional file 2:** R code used for haplotyping and statistical analysis.

## Data Availability

The datasets supporting the conclusions of this article are included in Additional File 1. Raw sequence data are available in the Sequence Read Archive repository, BioProject ID PRJNA826830 at http://www.ncbi.nlm.nih.gov/bioproject/826830.

## Abbreviations

*ama1-D3*: Apical membrane antigen 1
CHT: Chittagong Hill Tracts
*cox1*: Cytochrome C oxidase subunit 1
*cpmp*: Conserved *Plasmodium* membrane protein
*cpp*: Conserved *Plasmodium* protein
*csp*: Circumsporozoite surface protein
DNA: Deoxyribonucleic acid
H_e_: Expected heterozygosity
icddr,b: International Centre for Diarrhoeal Disease Research, Bangladesh
IBS: Identity-by-state
MOI: Multiplicity of infection
MS: Microsatellite
*msp1*: Merozoite surface protein 1
*msp2*: Merozoite surface protein 2
*msp7*: Merozoite surface protein 7
PCA: Principal component analysis
*pfhrp2*: *Plasmodium falciparum* Histidine rich protein 2
*pfhrp3*: *Plasmodium falciparum* Histidine rich protein 3
qPCR: Quantitative polymerase chain reaction
RDT: Rapid diagnostic test
*varATS*: *var* Gene acidic terminal sequence
